# The Impact of Senescence-Associated Secretory Phenotype (SASP) on Head and Neck Cancers: From Biology to Therapy

**DOI:** 10.3390/cancers17244024

**Published:** 2025-12-17

**Authors:** Md Tanjim Alam, Mishfak A. M. Mansoor, Sarah A. Ashiqueali, Pawel Golusinski, Ewelina Golusinska-Kardach, Joanna K. Strzelczyk, Blazej Rubis, Wojciech Golusinski, Michal M. Masternak

**Affiliations:** 1 Burnett School of Biomedical Sciences, College of Medicine, University of Central Florida, Orlando, FL 32827, USA; 2 Robert and Arlene Kogod Center on Aging, Mayo Clinic, Rochester, MN 55905, USA; 3 Department of Physical Medicine and Rehabilitation, Mayo Clinic, Rochester, MN 55902, USA; 4 Department of Otolaryngology and Maxillofacial Surgery, University of Zielona Gora, 65-417 Zielona Gora, Poland; 5 Department of Dental Surgery, Periodontology and Oral Mucosa Diseases, Poznan University of Medical Sciences, 61-701 Poznan, Poland; 6 Department of Medical and Molecular Biology, Faculty of Medical Sciences in Zabrze, Medical University of Silesia in Katowice, 41-808 Zabrze, Poland; 7 Department of Clinical Chemistry and Molecular Diagnostics, Poznan University of Medical Sciences, 61-707 Poznan, Poland; blazejr@ump.edu.pl; 8 Department of Head and Neck Surgery, Poznan University of Medical Sciences, 61-701 Poznan, Poland

**Keywords:** cancer biology, cellular senescence, DNA damage response (DDR), senescence-associated secretory phenotype (SASP), head and neck cancers (HNC), tumor microenvironment (TME)

## Abstract

Cellular senescence is defined as a state of permanent cell cycle arrest, providing a natural barrier against cancer. However, senescent cells are very metabolically active and secrete a complex mixture of bioactive molecules collectively known as the senescence-associated secretory phenotype (SASP), which play a dual role in cancer biology. While the SASP can suppress tumors by facilitating immunosurveillance, it can also promote tumor progression by fostering a pro-inflammatory milieu, stimulating angiogenesis, enhancing invasiveness, and enabling immune evasion. In Head and Neck Cancers (HNCs), a highly heterogeneous group of malignancies, SASP has emerged as a critical player in disease progression and treatment resistance. Persistent DNA damage response (DDR) signaling drives SASP and thereby contributes to the progression of head and neck cancer by modulating the tumour microenvironment. It influences the tumor microenvironment (TME) by facilitating epithelial-to-mesenchymal transition (EMT), promoting cancer stem cell-like properties, and impairing the efficacy of radiotherapy, chemotherapy, and immune checkpoint inhibitors. These effects underscore the need for targeted interventions to regulate SASP activity. This review presents a comprehensive overview of the molecular mechanisms underlying SASP generation and its effects on HNCs. We discuss the dual roles of SASP in tumor suppression and progression, its contribution to therapy resistance, and emerging therapeutic strategies, including novel senolytic and senomorphic drugs. Finally, we highlight key challenges and future directions for translating SASP-targeted therapies into clinical practice, emphasizing the need for biomarker discovery, and a deeper understanding of SASP heterogeneity. By targeting the SASP, there is potential to enhance therapeutic outcomes and improve the management of HNCs.

## Simple Summary

The senescence-associated secretory phenotype (SASP) plays a dual role in cancer by acting both as a tumor suppressor and a promoter of tumor progression. In Head and Neck Cancers (HNCs), SASP influences the tumor microenvironment, driving inflammation, angiogenesis, and therapy resistance while also contributing to immune surveillance. This review provides a mechanistic overview of SASP generation and its diverse roles in HNCs, highlighting how targeting SASP-related pathways could offer new opportunities for personalized and more effective therapeutic strategies.

## 1. Introduction

Head and neck squamous cell carcinoma (HNSCC), representing 90% of head and neck cancers, remains a pressing global health issue. Despite therapeutic progress, its incidence continues, especially among younger, non-smoking individuals. HNSCC remains a significant health concern in Poland, with an estimated incidence of around 6000 new cases annually. The mortality rate remains high, with approximately 50% of patients dying within five years of diagnosis, primarily due to late-stage detection [1,2]. Each year in the US, roughly 110,000 new cases of HNCs are diagnosed, resulting in about 17,000 deaths, and accounting for 4% of all new cancer diagnoses worldwide. In 2022, an estimated 1.9 million new cases of cancer and 609,360 cancer-related deaths were reported in the US. According to the 2025 ACS projections, approximately 66,000 new cases of oral cavity and pharyngeal cancers per year have been reported [3,4]. According to GLOBOCAN reports, Poland has one of the highest incidence and mortality rates of HNSCC in Europe, largely due to late diagnoses and high prevalence of tobacco and alcohol use [5]. Standard treatments include surgery, radiotherapy, and chemotherapy, often combined. Recently, immunotherapy has shown promise in recurrent/metastatic cases, improving survival in selected patients [6]. However, overall treatment response remains limited, and outcomes strongly depend on early diagnosis and HPV status. There is an urgent need for continued research because of the steadily increasing incidence and fatality rates linked to HNCs. To improve patient outcomes and survival rates, efforts must be made to create more accurate diagnoses, find novel medicines, and upgrade current treatment plans. Recently, immunotherapy has shown promise in recurrent/metastatic cases, improving survival in selected patients. However, overall treatment response remains limited, and outcomes strongly depend on early diagnosis and HPV status. HPV-positive HNSCC exhibits distinct biological and clinical characteristics compared to HPV-negative cancers, most notably a more favorable prognosis [7]. However, the risk of recurrence remains a significant concern. For HPV-positive oropharyngeal cancers, chemotherapy combined with radiotherapy is the standard of care. Patients with HPV-positive cancers often have a better prognosis and higher survival rate after radiotherapy/chemotherapy compared to patients with HPV-negative cancers [8]. The increased radiosensitivity of HPV-positive cancers is a key factor influencing better prognosis and potential modification of treatment strategies to reduce toxicity [9,10].

Cellular senescence refers to a state characterized by the permanent and stable arrest of the cell cycle, mediated by numerous stressors, including DNA damage, telomere shortening, and oncogenic signaling [11,12]. The ability of senescence to function as a natural defense against the formation of cancer by averting the development of damaged or potentially malignant cells led to its original identification as a tumor-suppressive mechanism. Senescent cells are not inert, though; they continue to function metabolically and release a wide range of chemicals that have a profound effect on their surroundings. These molecules impact the tumor microenvironment, often leading to progression of oncogenic transformation triggered by factors such as cytokines, chemokines, and other signaling molecules. Therefore, such a complex mixture of bioactive molecules is collectively known as the senescence-associated secretory phenotype (SASP) [13,14,15]. The SASP contains growth regulators, proteases, cytokines, and chemokines, which have the potential to modify the tissue microenvironment. It has been found that SASP links persistent DNA damage response (DDR) signaling to systemic immune responses. Despite the promotion of immune-mediated clearance of damaged cells, the SASP-DDR interaction may become dysregulated in the tumor microenvironment, contributing to tumor progression. Although the SASP can strengthen growth arrest and support the clearance of senescent cells, it may also drive tumorigenesis by inducing chronic inflammation, boosting angiogenesis, and altering the behavior of nearby cells [16]. This dichotomy underscores the complex role of the SASP in cancer and highlights the need to investigate the dual function of senescence as a crucial element influencing both tumor development and suppression [11,14,17,18,19].

Recent studies in the field of translation medicine have reported that by integrating senescence-inducing agents with immune modulation and targeted therapies, future holds great promise in the field of HNSCC management [20]. Considering the complex role played by SASP, research has highlighted the role of elevated mRNA expression of several SASP components in HNSCC tumor tissues compared with normal tissues, thereby correlating with disease progression. Furthermore, transcriptomic data have revealed a comprehensive SASP-related gene signature in HNSCC cohorts to predict patient prognosis and response to radiotherapy [21]. Mechanistically, therapy-induced senescence in HNSCC cells increases the release of SASP cytokines and chemokines, which then act on nearby cells to alter the tumor microenvironment, promote radioresistance, and support tumor survival.

Advancements in understanding the molecular mechanisms driving HNCs, particularly the influence of the tumor microenvironment and factors such as the SASP, are crucial. These discoveries may lead to the development of targeted treatments designed to combat treatment resistance and reduce the likelihood of recurrence [17,22]. Further investigation in this field may help mitigate the worldwide impact of HNCs and improve the lives of those affected. To identify potential treatment targets and improve patient outcomes, this review aims to elucidate the mechanisms by which SASP influences the initiation and progression of HNCs.

## 2. Cellular Senescence and SASP in Tumor Microenvironment

Senescence is a long-term halt in the cell cycle marked by morphological variations, chromatin and genetic modifications, metabolic shifts, and SASP secretion which includes cytokines, growth factors, and proteases. All of these inhibit the proliferation of aged and damaged cells which is closely linked to SASP-mediated regulation of the senescence program [23]. Intrinsic (oxidative damage and oncogene activation) and extrinsic (UV radiation, γ-irradiation, and chemotherapy) stresses trigger DNA damage responses (DDRs) that determine cell fate. Mild damage results in a temporary arrest, but severe damage triggers senescence or cell death processes, such as apoptosis, autophagy, or necrosis [24].

**Figure 1:**
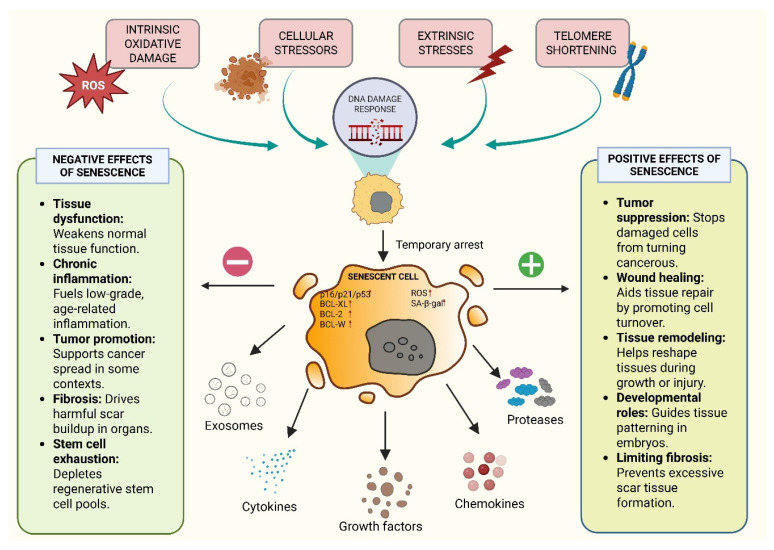
Overview of the Cellular Senescence Mechanism and Its Positive and Negative Effects.

Persistent DNA damage leads to activation of the DNA damage response (DDR). In this pathway, ATM kinase plays a major role in mediating DDR signaling at the centre, which further transcends genome repair and initiates inflammatory signaling [25]. Reports have suggested that chronic DNA damage triggers the association of ATM with the regulatory subunit NEMO. This results in post-translational modifications, including phosphorylation and ubiquitination, which promote the nuclear export of the ATM–NEMO complex to the cytoplasm, where it activates IKK, leads to IκB degradation, and allows NF-κB to translocate into the nucleus [26]. Activated NF-κB provides a connecting link between DDR signaling and the SASP-driven phenotype by mediating and triggering transcription of canonical SASP factors and other pro-inflammatory cytokines and chemokines, promoting a secretory phenotype from senescent cells. Thus, the DDR–immune crosstalk (DDR–ImmR) links genomic stress to a senescence-associated secretome. It helps to recruit myeloid and immune cells, providing a modality for tumor microenvironment changes and potentially facilitating tumor progression upon dysregulation [27]. Senescent cells exhibit persistent DDR signaling via the p53/p21 and p16INK4a/Rb pathways, and are in a non-dividing state. However, they are metabolically active and release SASP factors that substantially influence the TME (Figure 1). The figure provides an overview of the cellular senescence mechanisms along with its positive and negative effects and all the figures in this review are original scientific diagrams made by the authors [23,28].

### 2.1. Mechanisms of Senescence

**Figure 2:**
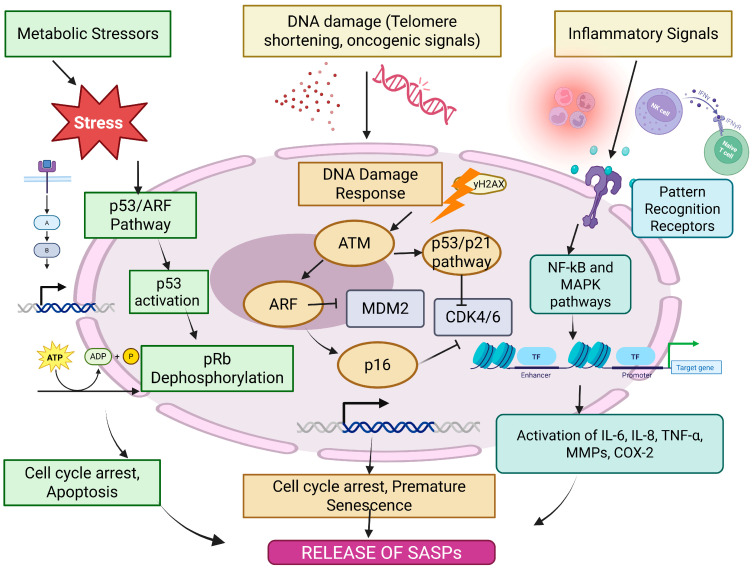
Mechanisms of SASP generation.

Key tumor suppressor pathways including p53/ARF and RB/p16, modulate the senescence state. DNA damage, a strong senescence inducer, triggers the DNA damage response (DDR) involving ATM, CHK1 and CHK2 [29]. p53, which transcriptionally regulates genes involved in cell cycle arrest and apoptosis, is phosphorylated and activated by these kinases [30,31]. Retinoblastoma (RB) dephosphorylation and activation can occur as a secondary mechanism when the DDR triggers p16 activity, consequently inhibiting CDK4/6. Cell cycle arrest is further reinforced by active RB [32]. The DDR is also triggered by telomere shortening, a major cause of replicative senescence. With every cell division, telomeres deteriorate until they become dysfunctional, activating ATM and γH2AX. Beyond the classical end-replication problem, telomeres are susceptible to oxidative damage, further accelerating cellular senescence [33]. Oncogene activation, especially by Ras, can also induce premature senescence through both DNA damage-dependent and independent mechanisms. It can also transcriptionally downregulate RRM2, affecting dNTP levels and further exacerbating DNA damage [34,35]. Therefore, the intensity and mechanism of SASP generation depend on the senescence-inducing insult, the cell type, and other various stress signals that collectively shape the local TME (Figure 2). The figure (original work) illustrates the complex network of cellular signaling pathways that lead to the establishment of the senescence-associated secretory phenotype (SASP) in response to various stressors. Metabolic stress, DNA damage (such as telomere attrition or oncogenic signals), and inflammatory stimuli activate pathways including the p53/ARF axis, p16/CDK4/6-mediated Rb dephosphorylation, and the DNA damage response via ATM kinase. These converge on cell cycle arrest and promote premature senescence. In parallel, pro-inflammatory signaling through pattern recognition receptors activates NF-κB and MAPK pathways, inducing the transcription of key SASP components such as IL-6, IL-8, TNF-α, MMPs, and COX-2. It has been found that RAS-activation triggers an oncogene-induced senescence which can orchestrate replication stress and downregulation of RRM2. The resultant dNTP pool depletion contributes towards activation of the DNA damage response which promotes SASP secretion. The consequent release of pro-inflammatory cytokines and chemokines remodels the tumor microenvironment. In head and neck cancer, oncogenes such as EGFR may interact with Ras-driven signaling pathways, linking oncogenic activation to paracrine SASP effects that support tumor progression [36].

### 2.2. Composition of SASP

**Figure 3:**
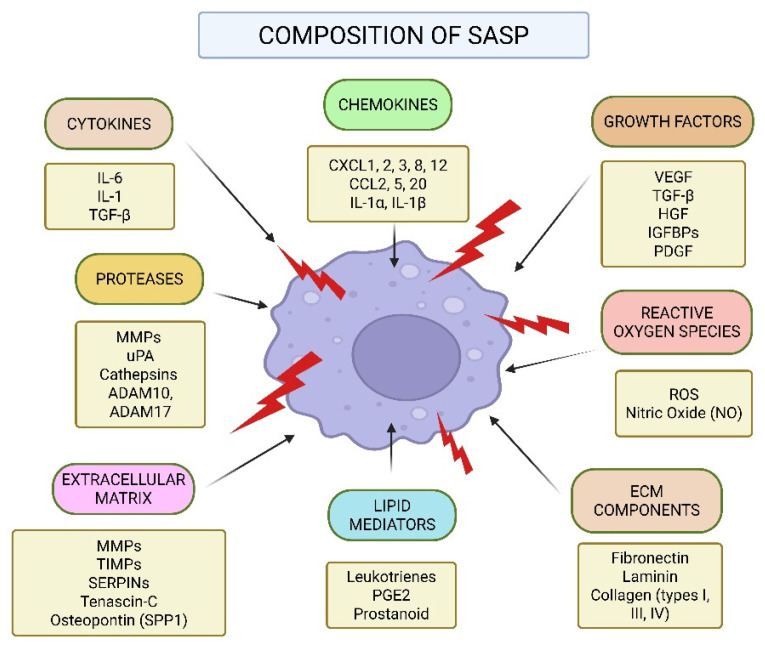
Composition of the Senescence-Associated Secretory Phenotype (SASP).

The SASP is composed of a unique mixture of molecules that influence the TME [17]. A summary of the various molecular elements that make up the senescence-associated secretory phenotype (SASP) is shown in Figure 3 (original work by authors). Cytokines, chemokines, growth factors, reactive oxygen species (ROS), extracellular matrix (ECM) modulators, and lipid mediators are among the complex mixture of pro-inflammatory and tissue-remodeling substances secreted by senescent cells. While chemokines like CXCL1-3, CXCL8, CXCL12, CCL2, CCL5, and CCL20, as well as interleukins like IL-1α and IL-1β, draw immune cells to the site of senescence, prominent cytokines like IL-6, IL-1, and TGF-β start and maintain inflammatory signaling [37]. Growth factors that affect angiogenesis, stromal remodeling, and cell proliferation include VEGF, TGF-β, HGF, IGFBPs, and PDGF. Furthermore, senescent cells produce nitric oxide and ROS, which strengthen DNA damage reactions and encourage paracrine senescence. Secreted proteases (e.g., MMPs, uPA, cathepsins, ADAM10, ADAM17), ECM structural elements (fibronectin, laminin, and different types of collagen), and regulatory molecules (TIMPs, SERPINs, tenascin-C, and osteopontin) all actively remodel the extracellular matrix. Moreover, local immune responses and inflammation are modulated by bioactive lipid mediators like prostanoids, prostaglandin E2 (PGE2), and leukotrienes [38]. These SASP elements work together to influence the tissue microenvironment, which has consequences for tumorigenesis, aging, and treatment resistance. Senescent cells release proinflammatory cytokines, such as IL-6 and IL-1, which play a crucial role in maintaining the state of senescence and influencing nearby cells through receptor-mediated pathways. Additionally, chemokines such as IL-8, Growth-Regulated Oncogene alpha (GROα), and Monocyte Chemoattractant Protein (MCP) family members contribute to immune cell recruitment and inflammatory signaling, thereby reinforcing growth arrest through self-sustaining feedback loops [39]. In addition to these proteins, the SASP includes numerous proteases, including matrix metalloproteinases (MMPs), serine/cysteine proteinase inhibitors (SERPINs), tissue inhibitors of metalloproteinases (TIMPs), and cathepsins (CTSs), all of which play a key role in breaking down and reshaping the ECM. This process is crucial for tissue remodeling and can facilitate cell migration [17,40,41]. Another major group of SASP factors includes damage-associated molecular patterns (DAMPs), such as high mobility group box 1 (HMGB1). DAMPs are released from a cell’s nucleus or cytoplasm when it experiences stress. These molecules act as alarms, triggering nearby cells into action. DAMPs, particularly HMGB1, help spread senescence signals, attract immune cells, and promote tissue repair [42,43]. In addition to proteins, senescent cells also release bioactive lipids that act as signaling molecules. These include eicosanoids, which are derived from arachidonic acid through enzymatic processes. Two key enzymes drive their production: cyclooxygenase (COX), which generates prostanoids, and arachidonate 5-lipoxygenase (ALOX5), which produces leukotrienes. These lipid messengers amplify inflammation and influence immune system activity within the TME. The insulin-like growth factor (IGF) mechanistic pathway is also involved in SASP, with senescent cells expressing elevated levels of IGF-binding proteins (IGFBPs), which influence cell proliferation and apoptosis [44]. Beyond proteins, senescent cells secrete non-protein molecules, including reactive oxygen species (ROS), nitric oxide, and lipid mediators like prostaglandin E2 (PGE2), which further modulate inflammation and cancer progression. In addition to signaling molecules, senescent cells secrete proteases that actively reshape the extracellular environment [45]. Matrix metalloproteinases (MMPs), particularly MMP-1, -3, and -10, degrade ECM components and regulate cytokine activity by cleaving chemokines like MCP-1 and IL-8. Serine proteases, including urokinase-type plasminogen activator (uPA) and its inhibitors PAI-1 and PAI-2, contribute to ECM remodeling and reinforce the senescence-associated growth arrest [46,47,48]. Furthermore, insoluble ECM proteins such as fibronectin are upregulated in senescent cells, affecting cell adhesion, migration, and tissue structure. Senescent cells also produce oxidative molecules such as ROS and nitric oxide, which can alter cellular metabolism, promote tumor progression, and accelerate aging-related degeneration. Collectively, these secreted factors enable senescent cells to profoundly influence their microenvironment, with implications for aging, chronic inflammation, and cancer development [49,50].

### 2.3. SASP and Cellular Communication

SASP promotes communication between senescent cells and their surroundings, influencing neighboring cancer and stromal cells. SASP alters the TME via attracting immune cells, altering stromal fibroblasts, and stimulating angiogenesis. SASP-induced IL-6 and IL-8 release has been shown to accelerate EMT in cancer cells, thereby enhancing their migratory and invasive capabilities [41,51]. Another study showed that therapy-induced senescence (TIS) in HNSCC is responsible for radioresistance by upregulating the components of SASP such as IL-6 and IL-8, which induce a pro-inflammatory and pro-survival phenotype [52].

SASP has both pro- and anti-tumorigenic activities on immune responses. Although senescence-associated cytokines in early-stage senescence enhance the immune-mediated clearance of senescent cells, the chronic senescence-associated secretory pathway (SASP) promotes immune evasion by recruiting immunosuppressive cell types. A study reported that in particular, elevated CCL2 and CXCL1 in senescent cells enhanced macrophage recruitment, resulting in an immunosuppressive microenvironment [18,19]. SASP recruits Tregs and MDSCs, reduces cytotoxic T lymphocyte activity, and inhibits anti-tumor immunity. Senescent cells can convert fibroblasts into cancer-associated fibroblasts (CAFs) via SASP-mediated paracrine signaling. CAFs then remodel the extracellular matrix and release more cytokines, resulting in a pro-tumorigenic TME. Studies show that SASP-induced fibroblast activation promotes tumor cell invasion and metastasis [53].

## 3. Role of SASP in Head and Neck Cancers

### 3.1. SASP and Tumor Suppression

In early tumorigenesis, SASP-driven immune activation plays a crucial role in clearing potentially malignant cells. For example, interleukin-6 (IL-6) and interleukin-8 (IL-8) recruit natural killer (NK) cells and macrophages, enabling the eradication of senescent cells before malignant transformation occurs [54,55]. Similarly, CXCL1 and CCL2 enhance the infiltration of monocytes and dendritic cells, reinforcing the tumor-suppressive effects of senescence [56,57,58]. Additionally, the SASP induces paracrine senescence in neighboring cells, effectively halting tumor expansion by amplifying the senescence response within the tissue. This non-cell-autonomous effect ensures that proliferative signals are confined to a restricted domain, preventing tumor progression. Another major tumor-suppressive mechanism involves the activation of the p53/p21 and p16^INK4a^/Rb pathways, which enforce cell cycle arrest and contribute to tumor suppression [11]. Persistent DNA damage signaling through the ATM/Chk2 axis further enhances SASP production, thereby reinforcing tumor suppression [59]. Recent studies have highlighted that SASP-driven immune activation is particularly effective in promoting tumor regression when combined with immunotherapy. For example, it was demonstrated that radiation-induced senescence triggers an SASP response that enhances T-cell permeation, leading to sensitization of tumors towards immune checkpoint barrier [60,61]. In a different study, Kang et al. demonstrated that long-term SASP-mediated immune surveillance causes hepatocellular carcinomas to produce a senescent response characterized by decreased tumor burden [62]. Additionally, it is known that angiogenesis inhibitor thrombospondin-1 (TSP-1) limits tumor vascularization and growth [63]. Prolonged SASP activity, however, can paradoxically aid in tumor progression through chronic inflammation and stromal remodeling, even though the SASP has robust tumor-alleviating effects in the early stages of cancer development [64,65]. Additionally, it was reported that SASP-derived IL-24 in hepatic cellular carcinoma attracts cytotoxic T-cells to eradicate premalignant cells, while senescent cells prevent tumor onset by activating p53-p21-Rb signaling [66]. Thus, preserving the positive effects of the SASP while reducing its pro-tumorigenic effects is a major difficulty in cancer treatment. To improve anti-tumor responses, senescence-inducing treatments like radiation and chemotherapy take advantage of SASP-mediated immune activation. However, research is still ongoing to determine which SASP components specifically support tumor suppression without escalating inflammation. When combined, SASP-induced tumor suppression is a basic process by which the body uses paracrine signaling, immune surveillance, and tumor-intrinsic barriers to prevent malignant transformation [11].

### 3.2. SASP and Tumor Progression

**Figure 4:**
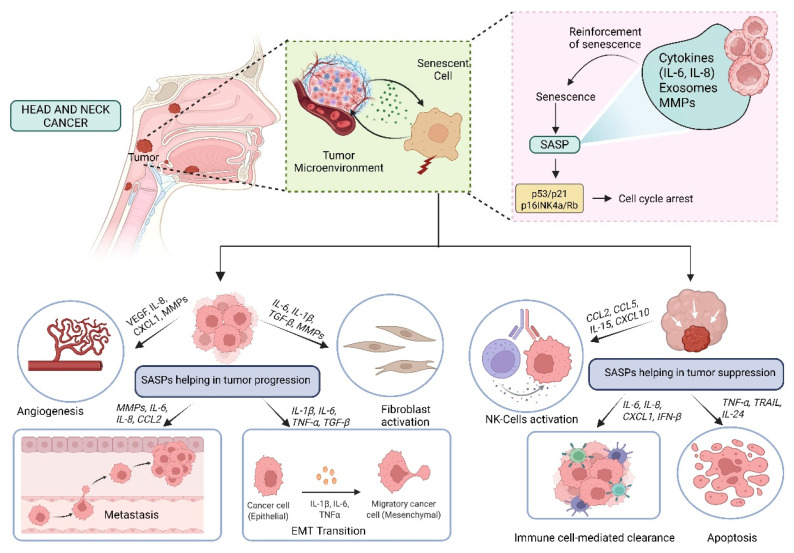
Role of SASP in Head and Neck Cancer: Tumor Promotion and Suppression.

SASP exhibits a paradoxical role in cancer, initially activating immune surveillance to clear malignant cells but later fostering a pro-tumorigenic microenvironment. This review highlights the dual nature of SASP in immune modulation—initially acting as a tumor-suppressive signal but eventually transitioning to an immune-evasive mechanism that sustains tumor growth. In the early stages, SASP components such as IL-6, IL-8, and CCL2 recruit immune cells to eliminate damaged cells, but prolonged SASP signaling alters this equilibrium, driving chronic inflammation, suppression of immune responses, and ECM remodeling, thereby facilitating tumor development [67]. One key mechanism driving this switch is the persistent activation of NF-κB and STAT3, transcription factors that regulate the expression of inflammatory cytokines and growth factors. In HNCs, it has been observed that SASP-induced IL-6 and IL-8 secretion promotes epithelial-to-mesenchymal transition (EMT), thereby increasing cancer cell motility and invasiveness [68]. For instance, IL-6 produced by senescent tumor-associated fibroblasts facilitated EMT and chemoresistance in oral squamous cell carcinoma (OSCC), a major subtype of HNCs, through activation of the JAK/STAT3 pathway [69,70]. Another hallmark of SASP-driven tumor progression is its role in ECM remodeling. Senescent fibroblasts secrete high levels of matrix metalloproteinases (MMPs), particularly MMP3 and MMP9, which degrade ECM components and facilitate cancer cell invasion. In laryngeal squamous cell carcinoma, overexpression of MMP-9 was linked with lymph node metastasis and poor survival, indicating its critical role in HNC progression [71]. Additionally, SASP factors such as TGF-β further promote fibroblast activation into cancer-associated fibroblasts (CAFs), which sustain a tumor-supportive microenvironment through continuous deposition of ECM proteins and secretion of additional cytokines that maintain inflammation [68,72,73]. A particularly insidious aspect of SASP-mediated tumor progression is its impact on angiogenesis. Numerous SASP components, including VEGF, IL-8, and CXCL12, have been shown to stimulate endothelial cell proliferation and neovascularization, facilitating the consistent transport of oxygen and nutrients to the tumor. A study in glioblastoma models demonstrated that SASP-driven VEGF secretion led to extensive vascularization, accelerating tumor expansion and resistance to anti-angiogenic therapies [74]. Similarly, in prostate cancer xenografts, targeting SASP-associated VEGF secretion using bevacizumab significantly reduced tumor vascularization and slowed progression [75]. Beyond angiogenesis, SASP also modulates immune responses in a way that favors tumor immune evasion. While early-stage SASP recruits immune cells for senescence surveillance, persistent SASP secretion eventually suppresses cytotoxic T-cell responses and promotes an immunosuppressive microenvironment [76]. In HNSCC, for instance, senescent tumor cells secrete elevated amounts of TGF-β and CCL22, promoting the infiltration of Tregs and impairing the ability of effector T cells to mount an effective anti-tumor response [77,78]. Another study using melanoma models showed that SASP-induced IL-1β release elevated PD-L1 levels in tumor cells, establishing an immunosuppressive barrier that diminished the effectiveness of checkpoint inhibitor therapies such as anti-PD-1 therapy [79] (Figure 4; original work by authors).

Despite scientific advances, clinical translation remains challenging due to the conglomeration of heterogeneous SASP factors across different tumor types and disease stages. Future research should focus on delineating SASP subtypes that drive specific aspects of tumor progression, allowing for more selective therapeutic targeting. Additionally, combining SASP-targeting strategies with existing immunotherapies and chemotherapy regimens may offer a synergistic approach to overcoming therapy resistance and preventing tumor relapse. The use of single-cell transcriptomics and proteomics will be crucial in identifying key SASP drivers in individual patients, paving the way for personalized interventions. While SASP initially functions as a tumor-suppressive mechanism, its prolonged activation creates a highly permissive environment for tumor growth, immune evasion, and therapy resistance [17,67]. Therefore, precise modulation of SASP—either by eliminating senescent cells with senolytics or selectively inhibiting pro-tumorigenic SASP factors with senomorphics—represents a promising avenue for cancer therapy. Nonetheless, additional research is required to comprehensively elucidate the context-specific impacts of SASP and to refine therapeutic approaches accordingly.

### 3.3. SASP and miRNAs in HNCs

**Figure 5:**
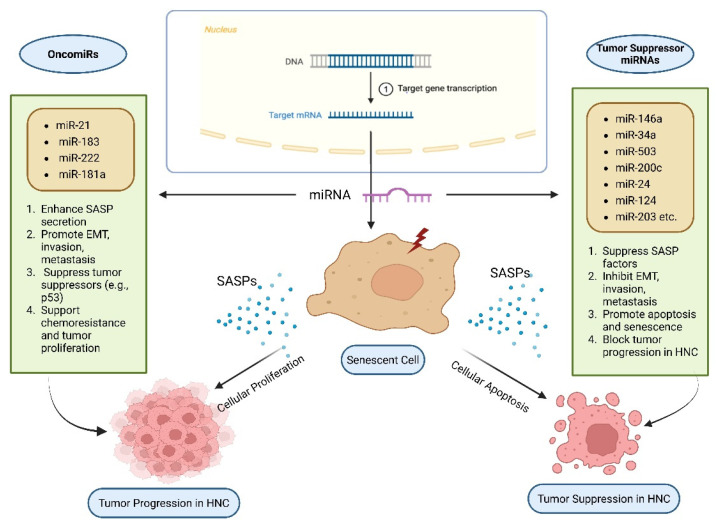
SASP and microRNAs (miRNAs) in Head and Neck Cancer (HNC).

MicroRNAs, or miRNAs, are short non-coding RNAs ranging from 18 to 20 nucleotides in length that play a crucial role in post-transcriptional gene expression regulation. When they bind to complementary sequences on target messenger RNAs (mRNAs), translation is inhibited [80]. In HNCs, miRNAs exhibit dual roles as tumor suppressors or oncogenes (also referred to as oncomiRs) by repressing cell proliferation and survival genes or silencing tumor suppressor genes (Table 1). They are found to regulate tumor formation and the SASP in HNCs (Figure 5; original work by authors). Numerous studies have demonstrated the role of miRNAs in influencing cellular senescence, inflammatory signaling, formation of SASPs, and subsequent effect on tumors [81,82]. For example, miR-146a has been shown to downregulate IL-6 and IL-8, the pro-inflammatory cytokines associated with SASP. They can also influence the NF-κB pathway and lead to a reduction of cytokine levels [83]. Another study demonstrated that miR-34a affects cellular senescence by decreasing the carcinogenic effects of SASP by targeting important mediators such as IL-6 [83,84]. oncomiR miR-21 has been found to be overexpressed in HNCs. It also blocks tumor suppressive pathways, along with worsening tumor-promoting SASP factors. Thus, it leads to promotion of cell survival and metastasis [85,86]. Additionally, miR-503 inhibits invasion and the epithelial-to-mesenchymal transition (EMT) by specifically targeting matrix metalloproteinases (MMPs) linked to SASP. Zinc Finger E-box Binding Homeobox 1 and 2 (ZEB1 and ZEB2) are frequently elevated by SASP factors such as TGF-β, and their downregulation is associated with greater invasiveness and metastasis in HNCs [87,88]. Other miRNAs, such as miR-200c, block EMT by targeting these transcription factors [89]. Additionally, miR-24 inhibits cell proliferation by downregulating genes linked to SASP, such as p16INK4a and p21, which are necessary for inducing senescence [90]. The functional impact of miR-24 is context-dependent. This can be attributed to the fact that the effects of miR-24 are influenced by the type and location of cells, the intricacies of the tumor microenvironment, and molecular signaling associated with the context. While downregulation of cell cycle inhibitors associated with miR-24 can alter senescence-associated signaling, it does not perpetually induce senescence. This demonstrates the complex and subtle function of miR-24 in regulating the equilibrium between senescence, cancer, and cell cycle progression. Moreover, miR-181a increases chemoresistance and cell survival by blocking p53, which indirectly increases the expression of SASP factor [91,92]. However, miR-124 suppresses tumors by inhibiting a crucial SASP regulator, STAT3 via targeting Bmi-1, a gene linked to stemness and SASP maintenance, miR-203 prevents tumor initiation and metastasis, while miR-125b affects senescence via controlling p53 and ERBB2, two factors that are implicated in the progression of SASP-associated tumors [93,94]. All things considered, these results imply that focusing on particular miRNAs may successfully alter SASP expression, providing a viable therapeutic strategy to lessen tumor aggressiveness and treatment resistance in HNCs [91].

## 4. Impact of SASP on Treatment Resistance in Head and Neck Cancers

**Figure 6:**
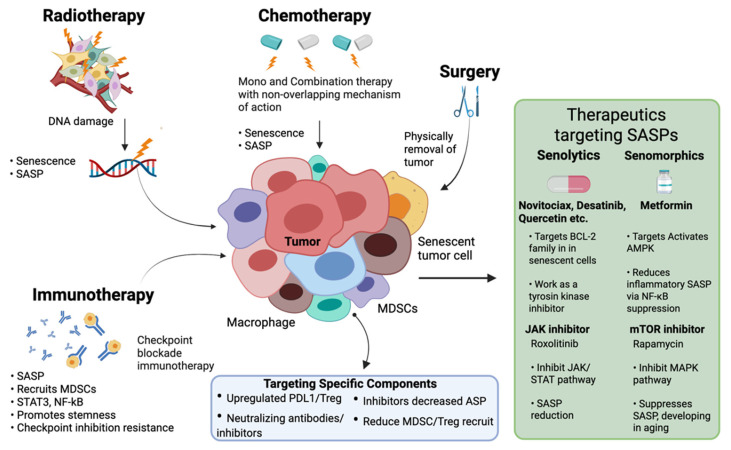
Therapeutic Strategies Targeting SASP in Head and Neck Cancer.

The SASP is a key regulator of the tumor TME and can impact treatment outcomes in HNCs. Although cellular senescence serves as a barrier against tumor development, the SASP paradoxically has the potential to drive tumor progression and foster resistance to therapy (Figure 6; original work by authors).

### 4.1. Radiotherapy and SASP

Radiotherapy is a cornerstone in HNC treatment, aiming to induce DNA damage leading to cancer cell death. However, sublethal radiation can induce senescence in tumor cells, resulting in the secretion of SASP factors. In addition, the inflammatory conditions generated by SASP factors facilitate the recruitment of immunosuppressive cells including myeloid-derived suppressor cells (MDSCs) and regulatory T cells (Tregs), ultimately impairing robust anti-tumor immunity. These SASP molecules also contribute to remodeling the tumor microenvironment by modulating immune cells, including macrophages, and potentially contributing to therapeutic resistance [19]. Several studies highlight the multifaceted role of cellular senescence and SASP in resistance to radiotherapy in HNSCC. In one such study, HNSCC cell line subclones with varying radiosensitivity were used and it was reported that radiation-resistant subclones exhibited increased SASP along with altered DNA repair and apoptosis [101]. Another study, also using HNSCC cell lines, investigated the IL-1 pathway and found that the expression of IL-1A and IL-1B strongly correlated with both radioresistance and senescence [102]. Lee et al. reported that SASP-related gene signatures predicted patient survival and were associated with radiation therapy (RT) response and role of SASP in resistance to RT in HNSCC. Another study focusing on mechanisms of radioresistance in HNSCC cell lines and xenografts identified a positive association between radioresistance and primary initiation of senescence. This senescence was linked to NF-κB-dependent production of specific SASP cytokines, which acted in a paracrine fashion to promote resistance. Importantly, inhibiting this cytokine production with metformin improved radiosensitivity [18,103]. Finally, a study focused specifically on the Fanconi anemia A (FancA) gene, located in a region previously linked to reduced progression-free survival after radiotherapy. They found that FancA overexpression conferred radioresistance in HNSCC cell lines associated with reduced interferon signaling, increased senescence, and elevated SASP. Critically, the clinical data confirmed that FancA amplification and overexpression correlated with poorer overall survival in HNSCC patients, further supporting the link between SASP, FancA, and radiation resistance [18,104]. Taken together, these studies suggest that the SASP plays a significant role in radioresistance in HNSCC and could be a target for future therapeutic interventions.

### 4.2. Chemotherapy and SASP

Chemotherapy is a key therapeutic approach for HNCs, frequently administered alongside surgical treatment. The chemotherapeutic drugs arrest cellular proliferation and are mainly involved in DNA damage induction and tumor cell apoptosis [9,105]. However, the role of SASP is often paradoxical in the context of chemotherapy for HNCs and deleterious since the SASP factors may lead to tumor recurrence and chemoresistance by promoting residual tumor cells’ survival and increased stemness [13,23]. For instance, it has been reported that in HNSCC, cisplatin treatment induces senescence, resulting in an SASP that promotes an immunosuppressive tumor microenvironment leading to chemoresistance. SASP factors also modulate the mechanisms of DNA damage and can cause the cancer cells to withstand chemotherapeutic-induced DNA damage lowering treatment efficacy. Cisplatin’s DNA-damaging properties can cause a strong senescent response in HNC cells, which aids in initial tumor suppression [28,106]. Nevertheless, the long-term influence of chemotherapy-induced senescence and SASP release is much more complex. Pro-inflammatory cytokine components of SASPs, such as IL-6 and IL-8, help in tumor cell survival, angiogenesis, and metastasis. They also stimulate signaling molecules downstream such as STAT3 and NF-κB, leading to therapy resistance and disease progression [41,51]. A study has shown that IL-6 can promote radioresistance in HNCs by activating the STAT3 signaling pathway. Furthermore, chronic inflammation from SASP factors recruits immunosuppressive cells hindering immune recognition and elimination of cancer cells post-chemotherapy [17,28]. For instance, it has been reported that immune checkpoint marker PD-L1 directly inhibit immune cell activity, thereby promoting immune evasion. Senescent cells can upregulate PD-L1 as part of a senescence-associated immune-evasion phenotype. The study showed that cisplatin-induced senescent HNC cells express high PD-L2, which reduces the efficacy of immune checkpoint blockade [22,107]. Additionally, SASP factors can also activate signaling pathways that promote drug efflux and DNA repair, leading to chemoresistance [108].

In conclusion, the role of SASP in chemotherapy for HNCs is complex and context-dependent. It is necessary to further investigate the complexity of interactions between chemotherapy, SASP, and the tumor microenvironment for effective therapeutic strategies. Future research should focus on targeted therapies and predictive target identification to develop a synergistic approach that overcomes therapy resistance and improves survival in HNCs.

### 4.3. Targeted Therapy and Immune Checkpoints

In the field of HNC therapeutic modalities, the current acceleration of research on immune checkpoint inhibitors (ICIs) has revolutionized translational science. They can be targeted to provide improvised and personalized therapies in combination with traditional approaches. The major challenge is the efficacy of ICIs and the inherent obstacles they present, as SASP factors are known to modulate molecules found at immune checkpoints, such as PD-L1, on tumor cells and within the TME, resulting in an immunosuppressive environment that reduces ICI efficacy [109]. Furthermore, the SASP’s pro-inflammatory cytokines can attract immunosuppressive cells, which further diminishes the immune system’s ability to fight tumors. SASP presents a particular feedback loop which encourages immunological evasion thereby strengthening the resistance to ICIs [110]. Additionally, SASP has also been found to influence the effectiveness of targeted medicines. For instance, treatment modalities get severely hindered due to the stimulation of alternative survival pathways in tumor cells, where SASP factors get actively released, resulting in reduced targeting of oncogenic pathways. Recent studies have further elucidated the complex role of SASP and the mechanical insights resulting in the modulation of the TME. Such processing ultimately influences the effectiveness of ICIs in HNCs [111].

Considering the studies in this sector, a study found that the presence of Tregs within the TME can aid in immune evasion in HNCs. Even though Tregs are known to suppress immune responses, it is important to understand their correct role in HNCs because their presence has been associated with both decreased and increased survival in various tumor types [112,113]. Another study investigated the varying reactions to ICIs in head and neck squamous cell carcinomas. The study found that very few HNSCC patients responded positively to ICIs whereas the majority showed no effect or inconsequential effect thereby suggesting the gaps in research and the possibilities of alternate pathways and SASP factors leading to the variability in treatment outcomes. Furthermore, a cohort study recently examined survival outcomes in patients receiving ICI-based treatment for recurrent or metastatic HNSCC. The study found that survival rates were similar to those reported in clinical trials and observed no significant difference in survival associated with stopping versus continuing immunotherapy at one or two years [114]. These findings underscore the need for personalized treatment strategies, considering factors like the SASP that may influence therapeutic resistance.

Altogether, these data divulge the multifunctional role of the SASP in establishing the TME and its implications on the therapeutic effectiveness of ICI in HNCs. They reinforce the requirement for further investigation into the complicated relationships among the SASP, immune regulation, and resistance to therapy in the quest to optimize the outcome of HNC therapy. In summary, while senescence induction in cancer cells was initially considered to be beneficial, the accompanying SASP has a detrimental effect by promoting therapeutic resistance in HNCs. It is essential to understand the SASP’s multifaceted role in shaping therapy responses, developing approaches to minimize its adverse effects while maximizing therapeutic efficacy.

## 5. Therapeutic Targeting of SASP

In HNCs, therapy-induced senescence, triggered by radiotherapy, chemotherapy, and targeted treatments, promotes chronic inflammation, immune evasion, and tumor progression. SASP factors, including IL-6, IL-8, and VEGF, reshape the tumor microenvironment (TME), promoting therapy resistance and metastasis [114,115]. Moreover, SASP-driven upregulation of immune checkpoints, such as PD-L1, weakens anti-tumor immunity, reducing the efficacy of immune checkpoint inhibitors (ICIs). Given these detrimental effects, therapeutic strategies targeting SASP are being explored (Table 2). Senolytic drugs are compounds that help eliminate senescent cells in a targeted and selective manner, while senomorphic agents modulate SASP production [116,117]. Additionally, inhibiting key SASP regulators, such as NF-κB and STAT3, offers a promising method. Overcoming these challenges may improve HNC treatment outcomes [118].

### 5.1. Senolytic and Senomorphic Drugs

Senolytics are compounds that selectively kill senescent cells by inducing apoptosis, thereby mitigating the harmful influence of SASP on the TME. Senomorphics are not cytotoxic to senescent cells; rather, they modulate the SASP to mitigate its unwanted pro-tumorigenic effects. Both approaches aim to mitigate the harmful effects of senescence in cancer treatment [116].

### 5.2. Potential Compounds and Their Relevance to HNCs

Multiple therapeutic strategies are currently being explored to target senescent cells and modulate SASP in head and neck cancers (HNCs). Senolytic agents like Navitoclax (ABT-263) induce apoptosis in therapy-induced senescent cells, improving treatment outcomes and potentially restoring normal tissue function [119,120,121,122]. Dasatinib and Quercetin, in combination, show promise in preclinical models by reducing systemic inflammation and senescent cell burden. With Quercetin, Dasatinib lowers the number of senescent cells in mice, reducing inflammation in adipose tissue and, improving systemic metabolic function [123,124,125]. JAK inhibitors, such as Ruxolitinib, function as senomorphics by downregulating SASP production via the JAK/STAT pathway and may improve immune responses in the tumor microenvironment [126,127,128]. Similarly, mTOR inhibitors like Rapamycin suppress SASP expression by modulating mTOR-ZFP36L1 signaling, reducing tumor growth and resistance in HNC models. mTOR inhibition has been associated with reduced tumor development and enhanced response to therapy, suggesting a role in SASP modulation for effective treatment [129,130,131,132] (Table 2).

**Table 2:** Potential Therapeutic Compounds and their relevance in HNCs.

Agent/Class	Mechanism of Action	Preclinical/Experimental Findings	Relevance to HNC/Other Models	References
Navitoclax (ABT-263)	BCL-2 family inhibitor; induces apoptosis in senescent cells (senolytic)	Eliminates therapy-induced senescent (TIS) cells; enhances response to chemotherapy; ablates senescent stem cells in irradiated salivary glands	Shown to reduce recurrence risk and improve salivary gland function in irradiated mice; reported benefit across cancer types, including HNCs	[128,129,130,131]
Dasatinib + Quercetin	Tyrosine kinase inhibitor + flavonoid; synergistic senolytic effect	Reduces number of senescent cells; lowers SASP; decreases inflammation in adipose tissue; improves metabolic function in mice	Combination therapy under investigation for enhancing efficacy of standard treatments and suppressing SASP-driven tumorigenesis	[132,133,134,135]
JAK Inhibitors (e.g., Ruxolitinib)	Inhibit JAK/STAT pathway; reduce SASP expression (senomorphic)	Modulate inflammatory SASP profile; reduce recruitment of immunosuppressive cells in the tumor microenvironment	Under evaluation in HNCs for restoring immune function and reducing SASP-mediated pro-tumorigenic signaling	[119,120,121]
mTOR Inhibitors (e.g., Rapamycin)	Suppress SASP by blocking mTOR-regulated translation of SASP transcripts via ZFP36L1	Inhibit MAPKAPK2-mediated ZFP36L1 phosphorylation, restoring SASP mRNA degradation; strong SASP suppressor	In HNC models, mTOR inhibition reduces tumor growth and enhances response to therapy by downregulating SASP	[122,136,137,138]

### 5.3. SASP Modulation Strategies

Currently, a promising research arena is understanding the SASP modulation strategies in HNC context so that the disease modalities and the therapy approaches can be refined for better patient outcomes. For SASP modulation strategies, one can either target specific SASP components and study their influence on the tumor microenvironment in relation with disease mechanics. Another way is to target upstream regulators and signaling pathways of SASP and inhibit those associated with tumor progression [128].

While targeting specific SASP components, it is necessary to identify key components of the SASP that promote tumor growth and immune evasion. For instance, key interleukins such as IL-6 and IL-8 play an important role in tumor progression. By interfering with their pro-tumorigenic signaling, neutralizing antibodies or small molecule inhibitors that target these cytokines, one can design approaches that can lessen their detrimental effects on the TME [133].

Similarly, research has found that the nuclear factor kappa-light-chain-enhancer of activated B cells (NF-κB) pathway is a critical regulator of SASP expression. It has been found that inhibitors of NF-κB signaling lead to decreased production of SASP factors and, in turn, influence the inflammatory signaling cascade [134]. They also influence the tumor-promoting activities within the TME by targeting SASP factors. Furthermore, signal transducer and activator of transcription 3 (STAT3) is another key regulator of SASP components. Targeting STAT3 with specific inhibitors can suppress the expression of pro-inflammatory cytokines and chemokines, potentially reducing tumor growth and enhancing the efficacy of ICIs in HNCs [135].

### 5.4. Challenges and Future Directions of Therapeutics in HNCs

To translate preclinical findings into effective clinical therapies targeting the HNC-associated SASP, a multitude of complex challenges must be addressed. One important challenge is the specificity of existing senolytic and senomorphic therapeutics. Even though these therapeutic agents aim to selectively eliminate senescent cells or modulate SASP production, off-target toxicity and injury to normal tissues continue to be a challenge. For instance, navitoclax and other senolytics target the BCL-2 family proteins, which are also present in certain normal cells, potentially leading to side effects such as thrombocytopenia. Enhancing the specificity of such medications, whether through tailored delivery systems or the development of more selective agents, is critical. A second significant difficulty is the variability of the SASP. The SASP’s constituents and quantities vary greatly depending on the cell type, inducing stimuli (e.g., chemotherapy, radiation), and microenvironment. Such variety prohibits the development of a “one-size-fits-all” approach to treating SASP. A tumor may contain a variety of senescent cells with varying SASP profiles, necessitating a tailored treatment plan. In addition, the SASP’s dynamic character, which varies over time and in response to therapy, adds another layer of intricacy [119,120]. To properly adapt treatments, we must track how the senescence-associated secretory phenotype (SASP) changes as HNC progresses. Finding precise markers to identify which patients will benefit most from SASP therapy is equally important. We currently do not have established markers to predict who may benefit from senolytic or senomorphic drugs. The lack of prognostic markers hinders the monitoring of treatment efficacy and makes it more challenging to recruit patients for clinical studies. An assay panel comprising biomarkers for the senescent cell load, SASP burden, and the state of relevant signaling pathways (e.g., STAT3, NF-κB) would be invaluable [121,122].

Another cause for concern is the potential of unintended consequences following SASP inhibition. While the SASP promotes tumor growth, it also aids in tissue repair and other physiological functions. Ablation of the complete SASP may thus have a deleterious impact on normal tissue function. A good technique focusing on manipulation of certain SASP components rather than total SASP ablation may be necessary. Finally, the efficacy of SASP-targeted therapy should be carefully assessed over time. Although preliminary preclinical findings are encouraging, we must observe whether reactions will last and result in long-term damage. To determine if these targeted medications successfully prevent recurrence and enhance survival for individuals with HNCs, long-term follow-up studies will be necessary [136,137].

The future of HNC therapies aimed at SASPs is to face the challenges posed by the existing problems and taking advantage of newly emerging research potential. One promising avenue is designing new senolytics and senomorphics with enhanced specificity and reduced toxicity. This will involve investigating novel chemical entities as well as drug repurposing from existing molecules with potential senolytic or senomorphic activity. Combining SASP-targeted agents with currently used therapies like ICIs, chemotherapy, or radiotherapy might improve therapeutic impact by preventing SASP-induced pro-tumorigenic activities and overcoming resistance processes [119,138,139]. Valid senescence- and SASP-related biomarkers can allow the stratification of patients and assessment of response to therapy, so that more efficacious and patient-tailored treatment schedules could be designed. The coordination between SASP-targeted therapy timing and conventional treatment may achieve optimized benefits [140]. For example, senolytic administration following chemotherapy-induced senescence can successfully eliminate senescent tumor cells and block relapse. The design of targeted delivery vehicles, like nanoparticles or antibody-conjugated structures, can make SASP-targeted agents more specific and less toxic, thereby increasing their survival in HNC patients [120] (Table 3). Senolytic agents, which selectively eliminate senescent cells, have shown promise in preclinical studies and early-phase trials, such as UNITY’s UBX0101 in osteoarthritis, although specific clinical data in HNCs remain limited. In parallel, combinatorial approaches that target the DDR–SASP–ImmR axis are being explored to simultaneously modulate DNA damage response signaling, attenuate SASP-mediated inflammation, and reshape the tumor microenvironment, potentially reducing tumor progression and therapy resistance.

## 6. Emerging Technologies and Models for Studying SASP in HNCs

It is evident that the SASP has a multifaceted role in Head and Neck Cancers (HNCs), regulating tumor growth and treatment response. Recent advances in research methods have provided new insights into SASP’s involvement in the tumor microenvironment, which must be thoroughly explored to pave the way for improved scientific possibilities [133,144].

### 6.1. In Vivo Models

To truly understand how the senescence-associated secretory phenotype (SASP) affects Head and Neck Cancers (HNCs), scientists employ two key experimental approaches. First, they utilize animal models, specifically patient-derived xenografts (PDXs). Here, human tumor tissue is transplanted into mice with weakened immune systems, allowing researchers to observe how the SASP influences tumor development and reactions to different treatments within a living organism [145]. This in vivo approach provides an understanding into the intricate relationship between SASP molecules and the surrounding tumor environment. However, PDX models present challenges, including high costs, modest transplantation success rates, and extended culture times. Recent advancements in cell culture techniques, particularly 3D organoids, offer alternative models that closely mimic in vivo tumor tissues. Second, three-dimensional (3D) organoid models have become crucial for studying the HNC tumor environment. These organoids, grown from patient tumor samples, are highly accurate representations of the original tumors, maintaining their structural and genetic characteristics [146,147]. This allows researchers to examine cellular interactions and the effects of the SASP on tumor behavior and treatment responses in a controlled laboratory setting. For instance, a study found that employing CRISPR-mediated gene editing via AAV to introduce human-relevant mutations directly into stem cells in vivo created a mouse model to study HNCs. Key findings include successful targeted mutagenesis across stem cell lineages, diverse mutation profiles in tumors, high mutational burden, evidence of precancerous states in adjacent tissue, identification of crucial gene targets for stem cell transformation, and a predominance of frameshift mutations, making it a valuable tool for studying HNC initiation and progression [148,149].

### 6.2. Organoid Models

Three-dimensional (3D) cell cultures that replicate the cellular and physiological tissue composition are called organoid models. They are stem-like cells that aggregate, self-organize, and develop into an organ tissue shape when cultured in 3D and are highly identical to their parent cells, with properties of self-renewal. Organoids can be used to simulate disease, screen medications, and even assess radiation-induced tissue responses, making them useful for research into the effects of radiation therapy on both normal and malignant HNC tissues. They can also be used in the field of personalized medicines and studying the SASP mechanisms in normal vs. HNC scenarios to get a better understanding of the therapeutic approaches and improvisation in this field. One particular study successfully generated organoids from 31 HNC patients. These organoids maintained the genetic and tissue-level characteristics of the original tumors, even over extended periods of time in culture, validating their reliability as representative models. Importantly, these organoids were able to predict how tumors would respond to cisplatin treatment, showing a strong correlation between in vitro drug sensitivity and patient outcomes. This suggests that organoids have significant potential for personalized medicine, enabling doctors to test different treatments on a patient’s tumor cells before administering them to the patient. In another study, the researchers reported a 30.2% success rate in establishing 3D cancer organoid cultures from patient HNC tissues. These organoid lines exhibited histopathological features similar to the original tumors and were utilized for in vitro and in vivo drug screening. The ability to replicate the tumor’s microenvironment makes organoids a promising platform for studying SASP’s role in tumor progression and therapeutic resistance [147,150,151,152].

### 6.3. Single-Cell Analysis

A thorough analysis of SASP’s function in HNCs is now possible thanks to developments in single-cell transcriptomics and proteomics. Single-cell RNA sequencing (scRNA-seq) has emerged as an effective method for investigating complex biological processes such as senescence and tumor metastasis. Using scRNA-seq, researchers discovered separate subpopulations of senescent cells with different gene expression profiles, including those linked with growth arrest, survival, and the SASP, as well as novel senescence programs involving long noncoding RNAs and splicing dysregulation [153]. Advancements in single-cell transcriptomics and proteomics have revolutionized the study of SASP in HNCs [147].

To illustrate the variability found in patient tumors, a study used bulk and single-cell RNA sequencing to identify genetic subgroups and intratumor heterogeneity in Patient-Derived Organoids (PDOs). Notably, they discovered a program resembling the hybrid epithelial–mesenchymal transition (hEMT) linked to poor patient survival and cisplatin resistance. The discovery of a senescence gene set (SenMayo) pertinent to various tissues and species has also been made possible by ScRNA-seq. This has enabled the identification of important intercellular signaling networks and characterize senescent cells at the single-cell level. In the context of cancer, scRNA-seq study of HNSCC identified a subset of pre-metastatic cells driven by actionable pathways, and indicated unique paths to T-cell failure, finding a role for SOX4. These findings underscore the importance of single-cell analysis in understanding SASP’s role in therapeutic resistance and tumor progression [154].

The integration of organoid models, single-cell analysis, and in vivo models has significantly advanced our understanding of the senescence-associated secretory phenotype in Head and Neck Cancers. These cutting-edge technologies provide comprehensive platforms for investigating the complex relationships within the tumor microenvironment, offering promising opportunities for personalized treatment and improved patient outcomes. These models will help clarify the complex role of SASP in tumor growth and treatment resistance as research advances, opening the door for new approaches to HNC treatment.

## 7. Future Directions of SASP in HNCs

The future holds immense promise given the importance of SASPs in HNCs. First of all, the current improved research and accelerated findings to identify key SASP-related biomarkers holds great promise in the field of diagnosis and prognosis in HNCs. Recent studies have identified gene signatures associated with SASP that correlate with patient outcomes, suggesting potential for these markers in clinical settings. SASP components, including IL-6, IL-8, TNF-α, and MMPs, are secreted in tumor microenvironments and can be detected in blood or saliva samples. It is critical to investigate the relationship between SASP molecule levels and clinical outcomes such tumor stage, therapy response, and survival. For example, studies have investigated the role of circulating IL-6 levels as a predictive marker in a variety of malignancies, including HNCs. Therefore, such studies highlight the future scope in the field of biomarker discovery for improved therapies of HNC patients utilizing the SASP milieu. The identification of robust and reliable SASP biomarkers could not only improve patient stratification for clinical trials but also enable personalized treatment strategies based on individual SASP profiles [122].

Secondly, use of personalized medicine approaches targeting SASP should be researched to create tailored approaches to potentially enhance treatment efficacy and overcome resistance by identifying the SASP factors to be targeted for tumor regression. This includes not just identifying patients who are most likely to benefit from SASP-targeted medicines but also personalizing the intervention to the characteristics of the individual tumor. Patients with malignancies that exhibit high levels of certain SASP components, such as IL-6 or IL-8, may benefit from targeted inhibitors of these cytokines in combination with standard chemotherapy or radiation. For instance, targeted inhibitors specific to cytokines showing elevated levels owing to SASP generation in patients with tumors may be used alongside the conventional radiotherapy or chemotherapy in HNC cases for better therapeutic outcomes [22,128,155,156].

Thirdly, the challenge of heterogeneity of SASP across different HNC subtypes needs to be addressed. Various factors such as HPV status, tumor location and physiology influence disease progression and response to therapy. There is a need to investigate the underlying mechanisms associated with SASP heterogeneity and then focus on developing targeted therapies to improve the efficacy of treatment modalities. Further research and standardized assays on SASP biomarkers, their detection, and target identification are needed to improve therapeutic outcomes in HNCs [122,156,157].

Lastly, the field of bioinformatics and sequencing holds tremendous promise in translational research. By using modern technological innovations and imaging techniques such as utilization of information from proteomics, single-cell analysis and imaging may aid in directing therapeutic choices. Single-cell proteomics and RNA sequencing pose significant promise in understanding the biology behind SASP heterogeneity at the cellular level and help in detecting unique SASP factors that contribute to tumor growth and therapy resistance. Additionally, three-dimensional organoid models provide a valuable platform for examining these interactions, as they accurately replicate the intricate cellular structure and architecture of tumors [147,150,151,152,158].

**Figure 7:**
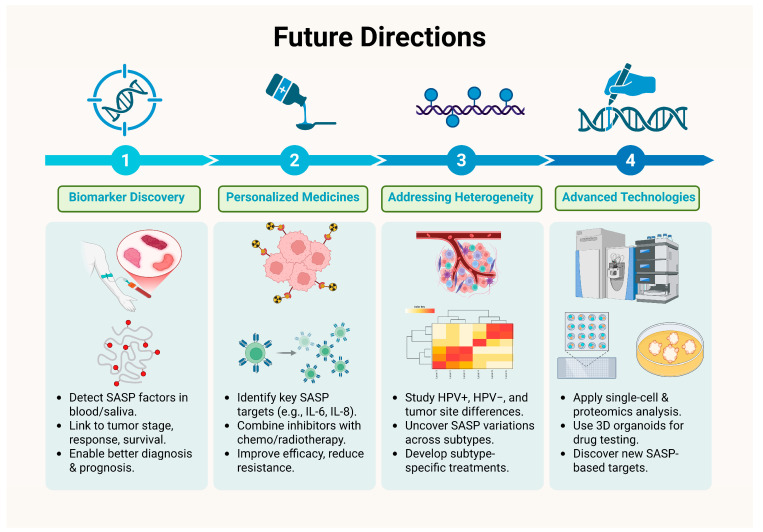
Future Directions in SASP related to Therapy in Head and Neck Cancer.

As a result, future research should focus on understanding the heterogeneity in SASP composition, its physiological and functional ramifications in various HNC subtypes, genetic as well as epigenetic factors, and the underlying mechanistic underpinnings, to develop more tailored and effective HNC treatments in the future [120,121,138]. Figure 7 (original work by the authors) efficiently summarizes the future directives in this regard.

## 8. Conclusions

In conclusion, the SASP can be considered a double-edged sword in Head and Neck Cancers. We have observed that through immune surveillance and accompanied growth arrest mechanisms, the SASP can inhibit tumor progression. Furthermore, its pro-inflammatory and pro-angiogenic activities can accelerate disease advancement, enhance stemness, and foster resistance to therapies.

Given the rising morbidity and mortality associated with Head and Neck Cancers, there is an urgent need to advance research on senotherapeutics. Approaches such as senolytics and senomorphics hold promises for improving clinical outcomes. However, the heterogeneity of the SASP and complexities of the tumor microenvironment pose significant challenges to the successful clinical application of these therapies [22,159].

Future studies should focus on identifying credible SASP biomarkers and understanding their function in various HNC subtypes to facilitate the development of personalized treatments. The dual effects of the SASP can be modulated to bring about revolutionary improvements in HNC management [81,159].

## Figures and Tables

**Table 1:** miRNAs in HNCs.

miRNA	Target Genes	Role in HNC	References
miR-146a/b	IRAK1, TLR8	Suppresses IL-6 and IL-8 expression, thereby lowering pro-inflammatory cytokines linked to SASP, and modulates the NF-κB signaling pathway.	[83]
miR-143	MMP13	Functions as a tumor suppressor associated with increased MMP13 expression, contributing to tumor invasion and metastasis.	[95]
miR-335	PTEN	Suppresses tumor formation by targeting PTEN contributing to tumor progression.	[96]
miR-183	ITGB1	Functions as an oncomiR, linked to increased ITGB1 expression, promoting tumor cell invasion and metastasis.	[97]
miR-9	IL-6	Regulates inflammatory signaling and SASP formation.	[20]
miR-222	MMP1	Functions as an oncomiR, overexpression is associated with increased MMP1 expression.	[89]
miR-125b	TNF, MMP13	Modulates TNF and MMP13, influencing inflammation and extracellular matrix dynamics.	[98]
miR-152	MMP3	Suppresses tumor invasion and metastasis by targeting MMP3.	[99]
miR-187	TNF, IL-6	Modulates inflammatory signaling and SASP by targeting TNF and IL-6.	[100]
miR-34a	IL-6	Affects cellular senescence by decreasing the carcinogenic effects of SASP by targeting IL-6.	[84]
miR-503	MMPs	ZEB1 and ZEB2 are frequently elevated by SASP factors like TGF-β, and their downregulation is associated with greater invasiveness and metastasis in HNCs.	[87]

**Table 3:** Therapeutic Targeting of SASP & Key Findings.

Therapeutic Approach	Mechanism of Action	Key Findings	References
Senolytic Drugs	Eliminate senescent cells by inducing apoptosis	Navitoclax (ABT-263), a Bcl-2 family inhibitor, has demonstrated potent senolytic activity by targeting anti-apoptotic pathways in senescent cells.	[141]
Senomorphic Agents	Suppress SASP secretion without killing senescent cells	JAK inhibitors (e.g., ruxolitinib) and mTOR inhibitors (e.g., rapamycin) have been shown to reduce SASP factors like IL-6 and IL-8, potentially mitigating pro-tumorigenic effects.	[125,127]
NF-κB Inhibitors	Block NF-κB signaling, a key regulator of SASP	Inhibition of NF-κB signaling has been found to decrease the expression of pro-inflammatory SASP factors, thereby potentially enhancing the sensitivity of cancer cells to therapies.	[134]
STAT3 Inhibitors	Inhibit STAT3 signaling, a major driver of SASP	Targeting STAT3 suppressed SASP-mediated immune evasion and improved response to immune checkpoint inhibitors in preclinical models.	[135]
IL-6/IL-8 Blockade	Neutralizing antibodies targeting IL-6 or IL-8	Blocking IL-6/IL-8 reduced inflammation-driven tumor growth and resistance to therapy in various cancer models.	[142]
p38 MAPK Inhibitors	Suppress SASP through p38 MAPK inhibition	Inhibition of p38 MAPK reduced SASP-driven inflammation and fibrosis, leading to improved treatment responses.	[143]
Immune Modulation	Enhance clearance of senescent cells by the immune system	Strategies like CD47 blockade improved immune-mediated senescent cell clearance in preclinical models.	[136]

## Data Availability

This study did not involve the generation or analysis of any datasets.
